# *Salmonella* invasion is controlled through the secondary structure of the *hilD* transcript

**DOI:** 10.1371/journal.ppat.1007700

**Published:** 2019-04-24

**Authors:** Chien-Che Hung, Colleen R. Eade, Michael I. Betteken, Paulina D. Pavinski Bitar, Elaine M. Handley, Staci L. Nugent, Rimi Chowdhury, Craig Altier

**Affiliations:** Department of Population Medicine and Diagnostic Sciences, College of Veterinary Medicine, Cornell University, Ithaca, NY, United States of America; University of California Davis School of Medicine, UNITED STATES

## Abstract

Virulence functions of bacterial pathogens are often energetically costly and thus are subjected to intricate regulatory mechanisms. In *Salmonella*, invasion of the intestinal epithelium, an essential early step in virulence, requires the production of a multi-protein type III secretion apparatus. The pathogen mitigates the overall cost of invasion by inducing it in only a fraction of its population. This constitutes a successful virulence strategy as invasion by a small number is sufficient to promote the proliferation of the non-invading majority. Such a system suggests the existence of a sensitive triggering mechanism that permits only a minority of *Salmonella* to reach a threshold of invasion-gene induction. We show here that the secondary structure of the invasion regulator *hilD* message provides such a trigger. The 5’ end of the *hilD* mRNA is predicted to contain two mutually exclusive stem-loop structures, the first of which (SL1) overlaps the ribosome-binding site and the ORF start codon. Changes that reduce its stability enhance invasion gene expression, while those that increase stability reduce invasion. Conversely, disrupting the second stem-loop (SL2) represses invasion genes. Although SL2 is the energetically more favorable, repression through SL1 is enhanced by binding of the global regulator CsrA. This system thus alters the levels of *hilD* mRNA and is so sensitive that changing a single base pair within SL1, predicted to augment its stability, eliminates expression of invasion genes and significantly reduces *Salmonella* virulence in mice. This system thus provides a possible means to rapidly and finely tune an essential virulence function.

## Author summary

Pathogenic bacteria tightly regulate the expression of their virulence functions to balance survival and proliferation within an animal host against the fitness costs that these functions engender. *Salmonella* has evolved an energetically favorable means to invade the intestinal epithelium, required for its virulence, with only a small proportion of its population expressing the needed genes, while the remainder reaps the benefits. This work shows that the threshold of invasion induction is finely controlled through the message secondary structure of the activator *hilD* in conjunction with the invasion repressor CsrA. This sensitive system may thus allow *Salmonella* rapidly to adjust the dynamics of its invading population in response to signals within the animal.

## Introduction

The success of bacterial pathogens relies upon a fine balance: They must rapidly induce functions dedicated to virulence in response to signals of the host, but withstand the often immense associated fitness costs that the production of these virulence proteins entails. This need is particularly acute for enteric pathogens, including *Salmonella*, as they survive within the intestine in competition with a vast number and diversity of bacterial species. Pathogens thus achieve this balance by several means. They may do so by evolving to place virulence under the control of existing global regulators. They may coordinate virulence gene expression as a part of tightly controlled, integrated regulatory mechanisms that respond to host signals. They may additionally induce virulence in only a select portion of a population sufficient to cause disease.

With its ability to survive within the host while also disrupting the intestinal mucosa to induce disease, *Salmonella* employs all of these techniques [1–3]. Invasion, the process of intestinal epithelial penetration, utilizes a type III secretion system to produce a multi-protein secretion apparatus [4]. The production of these structures, encoded within *Salmonella* pathogenicity island 1 (SPI-1) and including some forty genes, is controlled by a cascade of transcriptional regulators within SPI-1 comprised of HilD, HilC, HilA and InvF [5–8]. A host of global regulators outside the island have additionally been enlisted to control invasion [1,9–14]. Regulation of invasion has further been linked to that of metabolism through the BarA/SirA/Csr system [15,16]. The BarA/SirA two-component regulator induces two small regulatory RNAs, CsrB and CsrC. These RNA molecules in turn bind and titrate CsrA, a post-transcriptional regulator of both invasion and central carbon metabolism [17]. CsrA binds to the 5’ end of the *hilD* mRNA, sequestering the ribosome-binding site and start codon, and thus is proposed to prevent HilD translation [18]. The summation of these complex controls produces a state in which only a fraction of the *Salmonella* population is capable of invading tissue [3]. Invasion by this sub-population, however, alters the intestinal environment to favor the growth of luminal *Salmonella* [19–22], without imposing upon them the burden of virulence.

How then is this fine level of control achieved? The existence of two sub-populations, invasion-competent and -incompetent, suggests a threshold of genetic control: Any individual bacterium is capable of crossing that threshold, but only some minority of the total does. Here we show that such regulation can be achieved through post-transcriptional control of the *hilD* message in conjunction with the auto-induction of this central SPI-1 transcriptional activator. The *hilD* message employs alternative secondary structures, with mRNA stability enhanced by binding to CsrA, to control the production of HilD, and HilD in turn amplifies induction through the control of its own transcription. The sensitivity of this regulation is such that the addition of a single hydrogen bond within the message secondary structure completely prevents the expression of invasion genes and reduces virulence in an animal host. Thus, genetic and metabolic signals can be integrated to achieve the coordinated control of virulence.

## Results

### Activating mutations of *hilD* occur in its 5’ region

**Fig 1:**
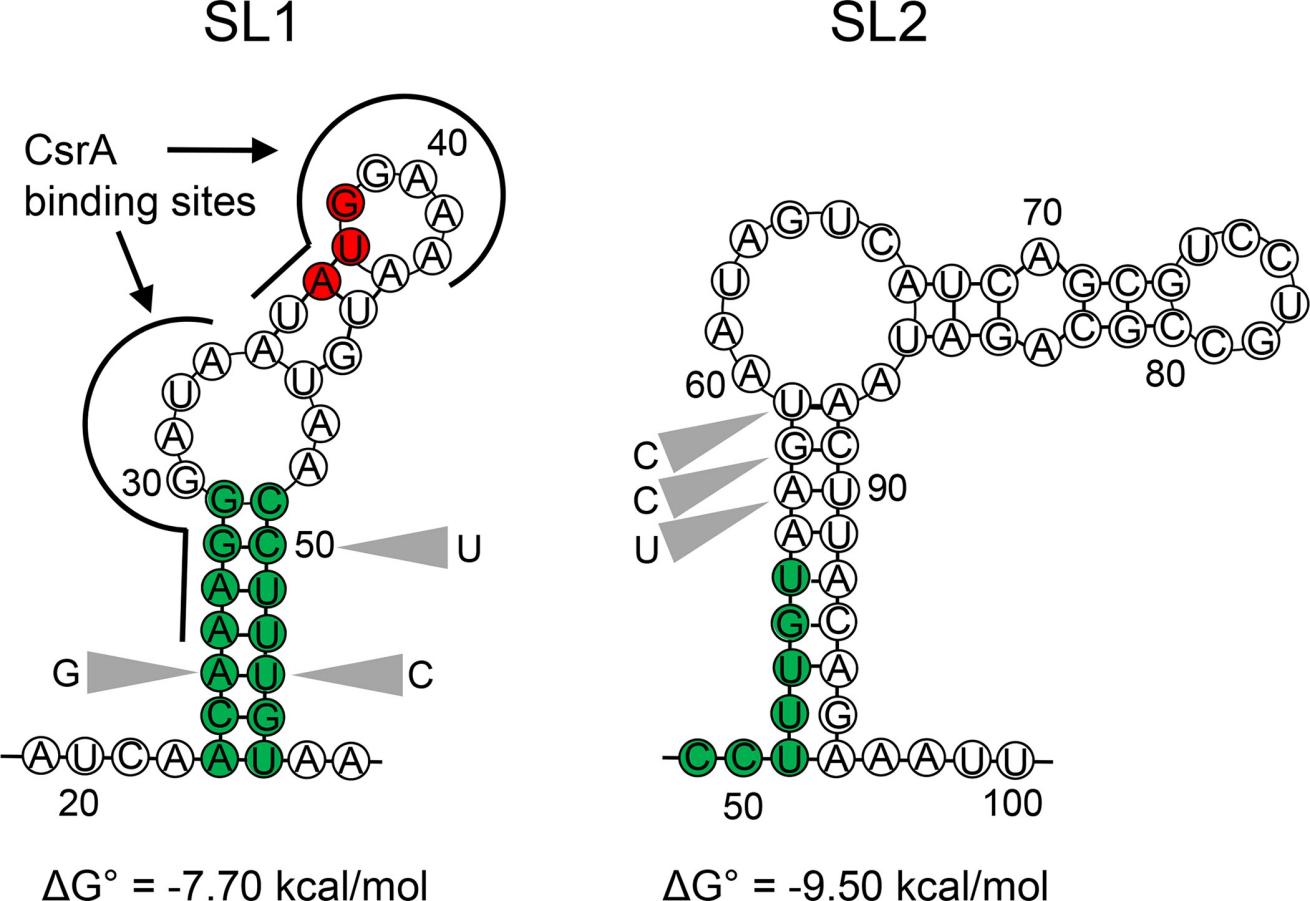
Predicted secondary structures within the *hilD* message. Bases shown in green represent base-pairs within stem-loop 1 (SL1) and their corresponding positions within stem-loop 2 (SL2). Bases in red indicate the *hilD* start codon. Grey triangles indicate the locations of mutations described in this study. The previously identified site of CsrA binding is shown by the black lines. Positions are numbered from the transcriptional start site.

The AraC-type transcriptional regulator HilD is known to be central to the control of *Salmonella* invasion, dictating the ability of the pathogen to penetrate the intestinal epithelium [6]. We sought to identify gain-of-function *hilD* mutants capable of inducing invasion gene expression under conditions in which HilD activity is greatly reduced and thus to identify *hilD* alleles with greater intrinsic expression or stability. We created these mutants using error-prone PCR on a plasmid construct that carried the *hilD* ORF and the portion of the 5’ untranslated region extending to the transcriptional start site, but replaced the native *hilD* promoter region with an inducible *tetA* promoter. The resulting plasmid library was introduced into a *hilD* null mutant harboring a transcriptional fusion of GFP to *sipC*, an invasion gene induced by HilD. Bacteria were grown in the presence of nonanoate, a fatty acid that strongly represses SPI-1 expression by reducing the stability of HilD protein (S1 Fig), and mutants with aberrantly high expression of GFP were identified. This screen identified a cluster of mutations within the 5’ untranslated region and extending into the 5’ end of the *hilD* ORF that provided gain of *hilD* function. These mutations occurred at nucleotides +23 to +31 and +46 to +53, as numbered from the transcriptional start site (S2 Fig). By analyzing minimal free energy, we found that the transcript in this region is predicted to form a stem-loop secondary structure (termed here Stem-Loop 1; SL1) with a free energy of -7.70 kcal/mol, having seven perfect nucleotide pairs at its base, and incorporating the initiating codon of HilD at position +36 (Fig 1). The mutations identified lie entirely within SL1 and largely within its predicted stem structure.

### Changes in *hilD* message secondary structure affect *Salmonella* invasion gene expression and virulence

**Fig 2:**
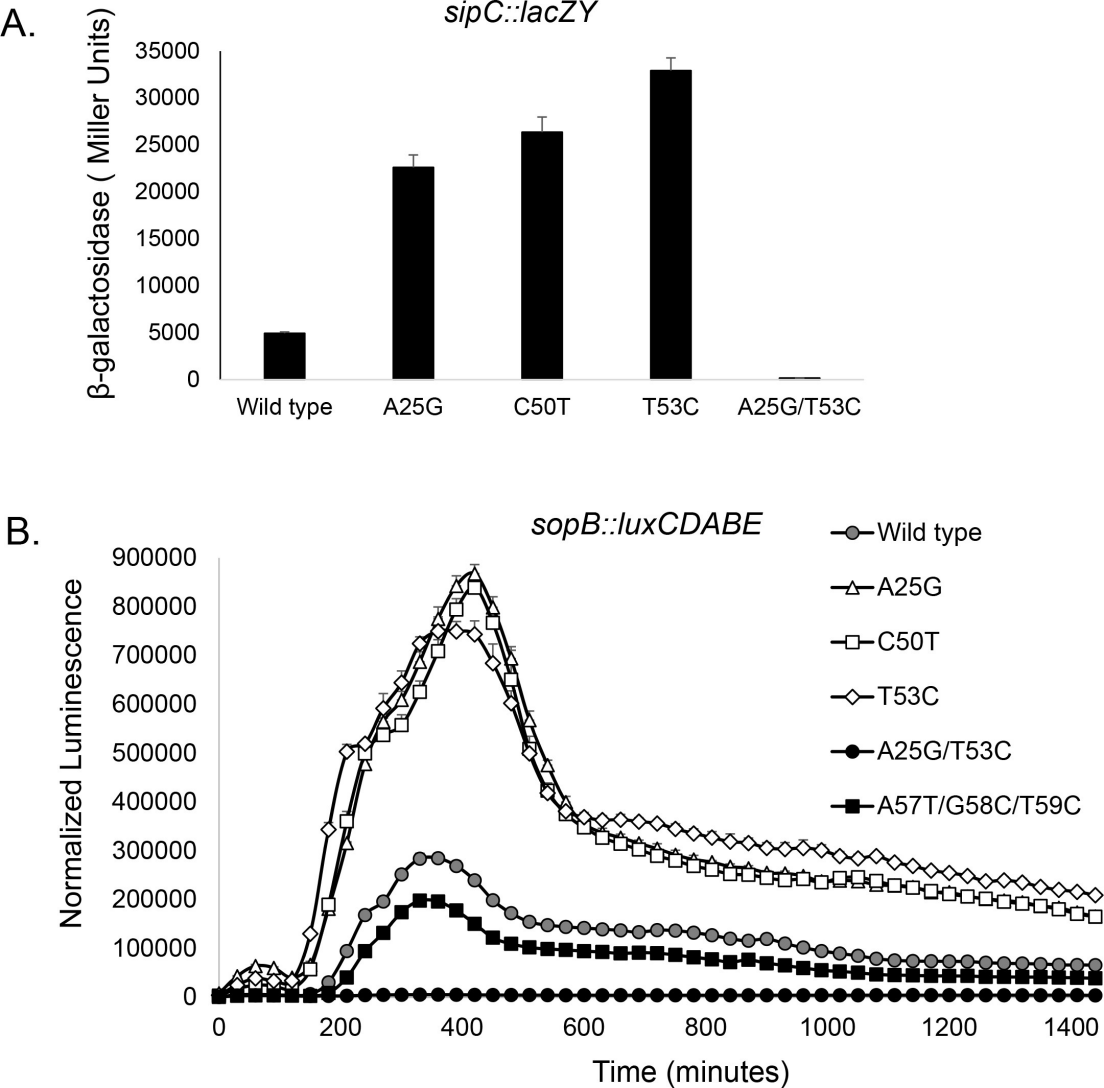
Mutations affecting the *hilD* message secondary structure alter invasion gene expression. (*A*) Expression of the invasion gene *sipC* was assessed in the mutants shown using a *lacZY* transcriptional reporter fusion by β-galactosidase assays. Histograms and error bars represent means ±SD (n = 3). (*B*) Expression of the invasion gene *sopB* was measured over time using a *luxCDABE* transcriptional reporter fusion (n = 5), assessing luminescence normalized to bacterial numbers (luminescence/OD_600_). All strains differed from the wild type for (*A*) mean expression at P < 0.001, and (*B*) mean peak expression at P < 0.0001.

To examine the importance of this region to invasion, we created chromosomal point mutations of specific bases. The resulting mutants, created using CRISPR/Cas9, carried single, unmarked mutations without additional genetic changes to the region. The change of A25 to G, C50 to T, or T53 to C, all predicted to disrupt base pairing within the SL1 stem (Fig 1), greatly increased expression of invasion genes. We tested a single-copy chromosomal *sipC*::*lacZY* reporter fusion as a representative HilD-regulated gene within SPI-1, and found it to be increased by 4- to 6-fold in these mutants (Fig 2A). A plasmid-borne *sopB*::*luxCDABE* was additionally tested as a HilD-regulated gene outside of SPI-1, showing peak expression of the mutants increased by >2.6-fold and areas under the curve increased by at least 2.7-fold (Fig 2B). The expression of *hilD* itself, known to be auto-induced, was similarly affected by these mutations, with at least two-fold increases in expression in the mutants (S3A Fig). Neither of the mutations created within the *hilD* ORF (C50T or T53C) altered the HilD amino acid composition, thus eliminating any possible effects by HilD protein and instead implicating the mRNA transcript as the source of these effects.

**Fig 3:**
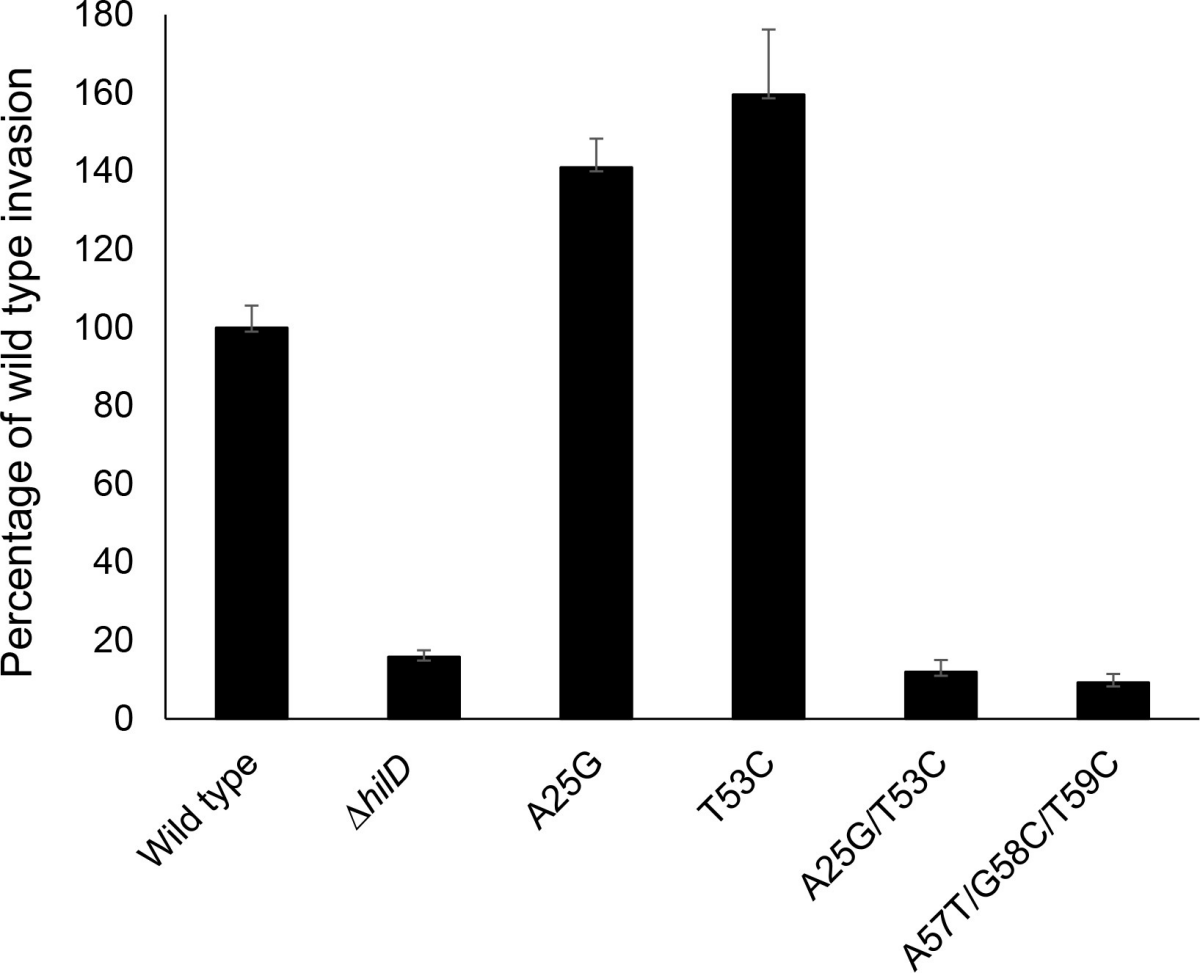
Mutations affecting the *hilD* message secondary structure alter invasion of cultured cells. The ability of strains to penetrate cells was determined using cultured HEp-2 cells in a gentamicin-protection assay. Invasion is shown relative to the wild type set to 100%. Data show mean ±SEM for the combined results of two independent experiments, each with four replicates (total n = 8 for each strain). Invasion of all mutant strains differed from that of the wild type at P < 0.001.

To confirm the importance of this region to the regulation of invasion, we further tested a compensatory double mutant, carrying both the A25G and T53C changes. This mutant is predicted to change an A-U base pair within the message to G-C, reducing the predicted free energy to -9.50 kcal/mol, and thus potentially enhancing secondary structure stability. Indeed, the expression of invasion genes in this mutant, in contrast to the induction in the single mutants, was vastly repressed compared to that of the wild type, with expression of *sipC* reduced by 29-fold and *sopB* by 51-fold. (Fig 2). These mutations similarly altered the ability of mutant strains to penetrate cultured epithelial cells, demonstrating their functional significance. Mutants of A25G or T53C showed significantly increased invasion of HEp-2 cells compared to the wild type, by 1.4- and 1.6-fold respectively, using a gentamicin-protection assay (Fig 3). In contrast, the A25G, T53C double mutant invaded as poorly as did a *hilD* null mutant, reduced from the wild-type level by 8-fold. Thus, this single change to a predicted pair of bases within this region of the *hilD* message, increasing hydrogen bonding by one, fully abrogated invasion gene expression and epithelial cell invasion.

To directly test the effects of these *hilD* mRNA point mutations on invasion *in vivo*, we employed the murine typhoid fever model to measure their colonization of abdominal organs, a function that requires invasion. *Salmonella*-susceptible BALB/c mice (*Slc11a1* negative) were inoculated orally with similar numbers of two strains, the wild type and the *hilD* A25G, T53C double mutant that demonstrated greatly reduced invasion gene expression in laboratory media. Numbers of each strain in the spleens and livers of infected mice were assessed five days post-inoculation. We found that, in accordance with our gene expression studies, the A25G, T53C double mutant reached these tissues only poorly. The mean competitive index of the two strains, defined as the number of wild-type bacteria divided by that of the mutant, was 651-fold in the spleen (P = 0.049) and 322-fold in the liver (P = 0.020), demonstrating a significant deficit in invasion by the mutant, and indicating the essential nature of this region to *Salmonella* virulence. The single mutants tested, however, (A25G or T53C) did not demonstrate consistent enhanced virulence but instead showed erratic behavior, with competitive indices to the wild type varying greatly among individual mice (S4 Fig). This may be due to the metabolic burden caused by the over-expression of invasion genes in these mutants, manifested by reduced growth rates *in vitro* (S3B Fig), or by the dysregulation of virulence genes once invasion has been accomplished within the host, thus compromising bacterial survival within tissues.

### Alternative transcript secondary structure alters invasion gene expression

Because these *hilD* mutants, predicted to affect only single base pairs within the message, manifested such profound effects on invasion, we further examined the area adjoining the SL1 secondary structure for regions that might affect its formation. mRNA structure analysis indicated the possibility of a second stem-loop in the *hilD* transcript (SL2), located within the 5’ region of the *hilD* ORF. Its stem portion consisted of nine pairs of nucleotides, of which eight form perfect base pairs, and was predicted to have a free energy of -9.50 kcal/mol, more favorable than that of SL1 (Fig 1). This structure, at positions +51 to +96, would share five nucleotides with the stem portion of SL1, making the simultaneous existence of the two impossible. We thus hypothesized that SL2 provides an alternative transcript conformation that counteracts the repression of invasion gene expression imposed by SL1. To test this, we created mutations designed to disrupt the predicted stem portion of SL2 (Fig 1). As these exist within the *hilD* ORF, synonymous mutations were again created to alter nucleotides without changing the amino acid composition of HilD. The mutations A57T, G58C and T59C together disrupt three base pairs within SL2 and predict a change in the free energy of SL2 from -9.50 kcal/mol to -7.20 kcal/mol, thus potentially making this mRNA conformation less favorable to that of SL1. We found that such a triple mutant demonstrated significantly decreased invasion gene expression when compared to the wild type (Figs 2B and 5A and S4A Fig), and reduced epithelial cell invasion to a level indistinguishable from that of a *hilD* null mutant (Fig 3), indicating an effect opposite to that of SL1, and suggesting the importance of alternative mRNA secondary structures within the *hilD* message for the control of invasion.

### Mutations in the *hilD* message alter transcript levels independently of transcription in association with the regulatory protein CsrA

**Fig 4:**
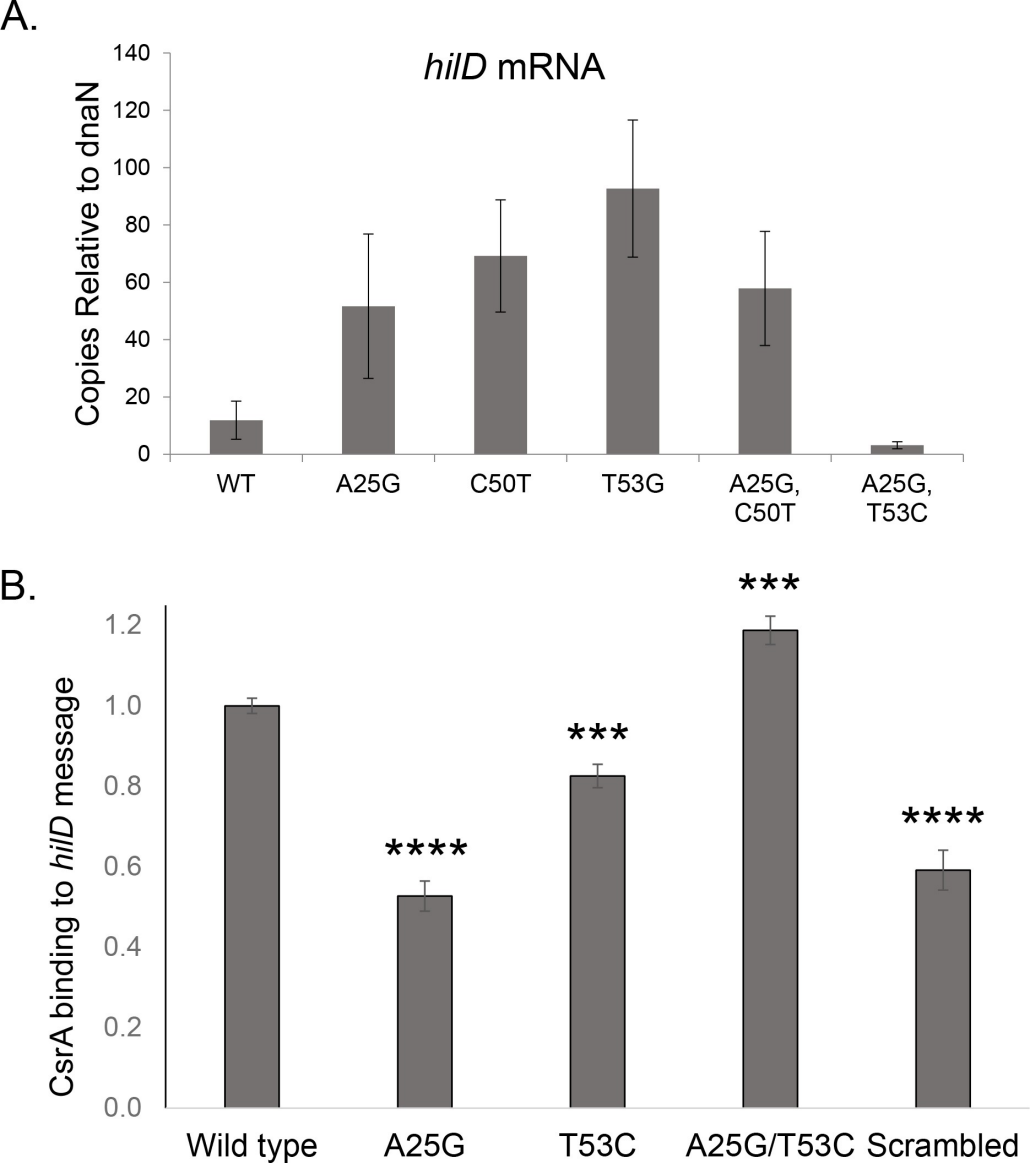
Conformation of the *hilD* mRNA alters message stability through binding of SL1 to CsrA. (*A*) Mutations within the *hilD* mRNA alter message stability. Strains of the genotypes shown and with *hilD* removed from its native expression by fusion to a *tetA* inducible promoter were assessed by RT-PCR for expression of *hilD*. Copies of *hilD* message are shown compared to *dnaN*. Histograms show mean ±SD for three independent experiments each performed in duplicate (total n = 6). All mutants differed from the wild type at P < 0.05. (*B*) Mutation of the *hilD* mRNA alters message binding to CsrA. Purified his-tagged CsrA was incubated with 5’-biotinylated RNA probes of the sequence shown, and RNA-protein binding was assessed by blotting the mixture onto nitrocellulose membranes. Binding is shown relative to the wild type, set to 1. The scrambled probe, a randomly generated sequence of the same length and G-C content as the other probes, served as the control. Data show the combined results of three independent experiments, each with five replicates (total n = 15). Histograms show means ±SEM. ***P < 0.001;****P < 0.0001.

The importance of SL1 to invasion, in spite of the presence of the competing SL2 with its predicted greater stability, suggests the existence of additional means to stabilize transcript topology. It has previously been reported that the regulatory protein CsrA binds to two sites on the *hilD* mRNA, at positions +26 to +33 and +35 to +42 [18], both located within SL1 (Fig 1). CsrA is a component of the BarA/SirA/Csr regulatory cascade that integrates invasion and metabolic control, and inhibits invasion by the post-transcriptional repression of *hilD*. This regulator has been shown to function by preventing translation and reducing message half-life of its target genes [23]. We thus hypothesized that CsrA might recognize its binding sites in the context of SL1, enhancing stability of this inhibitory secondary structure, and therefore that disruption of SL1 would promote the accumulation of *hilD* message. To test this, we expressed wild type and SL1 mutant alleles of *hilD* from an exogenous, inducible promoter (P_*tetA*_), removing its native transcriptional control, and assessed message levels using RT-PCR. The presence of A25G, C50T, or T53C mutations all significantly increased the concentration of *hilD* message, by 4- to 8-fold compared to that of the wild type (Fig 4A). As transcription is expected to be invariant in these strains, the results suggest that the increases in message levels are due to the improved stability of the *hilD* transcript, consistent with a predicted reduced function of CsrA in these mutants. Message levels were, conversely, decreased in the A25G, T53C double mutant, suggestive of enhanced efficacy of CsrA and consistent with its defect in invasion.

To test directly the interaction of SL1 with CsrA, we measured the ability of CsrA to bind to wild type and mutated RNA fragments of this region. Biotinylated RNA probes consisting of the SL1 region alone were incubated with purified CsrA protein, transferred to nitrocellulose membranes, fixed with UV light, and quantitated using HRP-conjugated streptavidin. Small RNA molecules are unable to bind efficiently to nitrocellulose and thus will do so only if associated with protein. We found that the A25G mutation reduced CsrA binding of SL1 to 53% (P < 0.0001) of the wild type level, indistinguishable from the binding of CsrA to a scrambled RNA (randomly generated with the same length and G-C content as SL1, but without discernable secondary structure) (Fig 4B). The effect of the T53C mutation was not as strong, but significant, with CsrA binding reduced to 82% (P < 0.001) of the wild type level. Conversely, the A25G, T53C double mutation increased binding to 119% (P < 0.0001). These results thus show that changes that reduce SL1 stability, A25G or T53C, also reduces CsrA binding, and one that enhances that stability, A25G, T53C, has the opposite effect.

**Fig 5:**
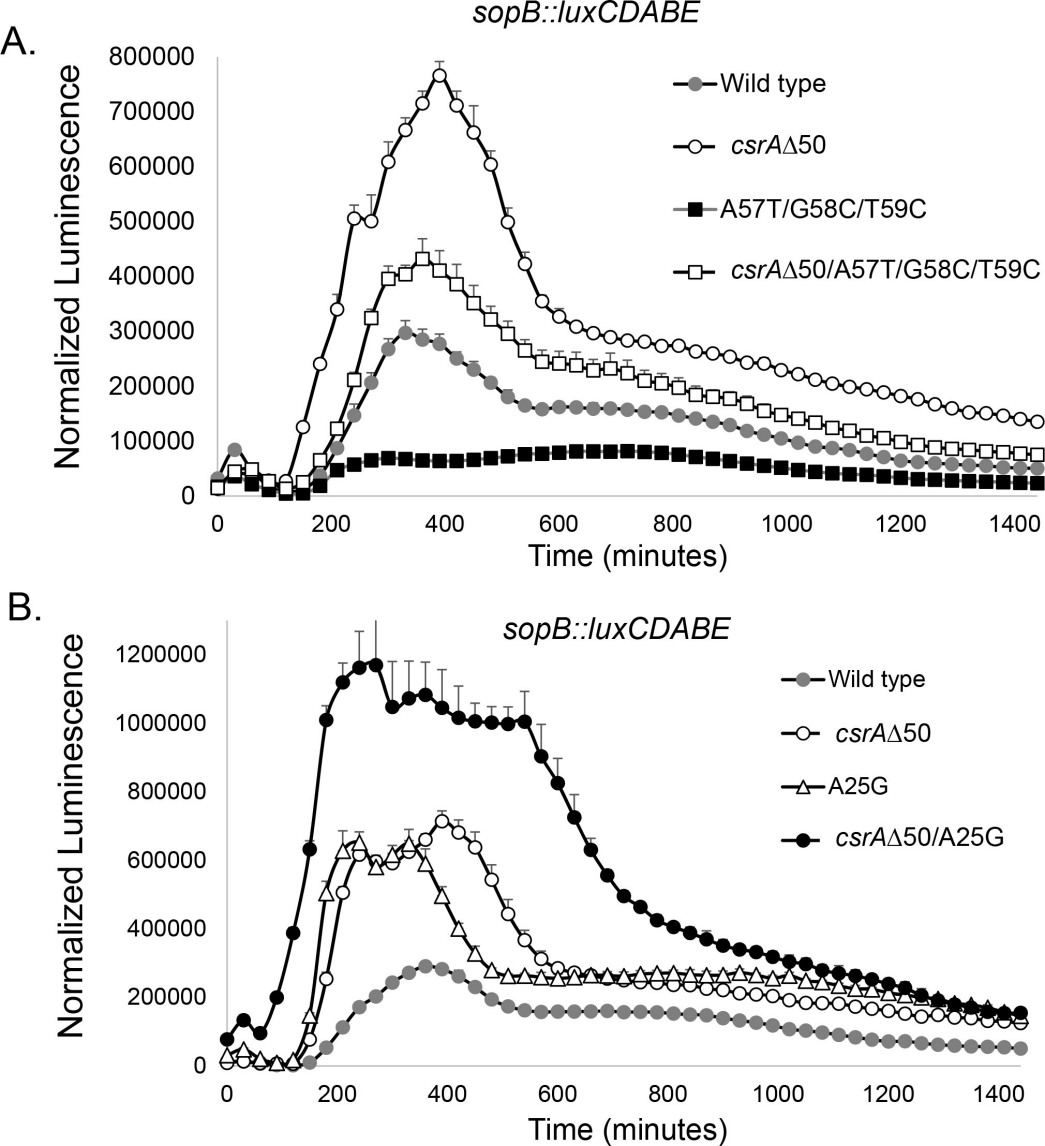
CsrA and *hilD* message secondary structure coordinate the control of invasion gene expression. Expression of the invasion gene *sopB* was determined in the strains shown using a *luxCDABE* transcriptional reporter fusion and measuring luminescence normalized to bacterial numbers (luminescence/OD_600_). (*A*) Repression of invasion gene expression by disruption of SL2 is countered by the loss of CsrA function, indicating reciprocal activities of the two. Data show mean ±SD (n = 4 for each strain). Peak expression of all strains differed from that of all other strains at P < 0.0001. (*B*) Combined disruption of SL1 and CsrA has additive effects on invasion gene expression, indicating a retained partial function of SL1 in the absence of CsrA and of CsrA in the absence of intact SL1. Data show mean ±SD (n = 4 for each strain). Peak expression of all strains differed from that of the wild type, and the *csrAΔ*50, A25G double mutant from that of either single mutant at P < 0.0001.

To examine the consequences of these interactions with CsrA, we next tested the effects of a *csrA* mutation on invasion gene expression in the wild type and secondary-structure mutants. Strains with deletions of *csrA* grow very poorly, and so we instead used a truncated mutant lacking the 11 amino acids of the carboxyl terminus, reducing its size to 50 amino acids (*csrAΔ*50). An equivalent mutant has long been successfully employed in *E*. *coli*, with its identical CsrA protein, and demonstrates defects in multiple CsrA-controlled pathways that indicate impaired regulatory function by this truncated protein [24]. As anticipated, the *csrAΔ*50 mutant increased *sopB* expression in an otherwise wild type strain, demonstrating that it is unable to effectively repress invasion (Fig 5). We first tested the activity of CsrA in an SL2 mutant. If CsrA augments the stability of SL1, thus favoring this secondary structure over the competing SL2, one would predict the loss of CsrA to restore invasion gene expression in an SL2 mutant. Indeed, we found this to be the case: the A57T, G58C, T59C triple mutant of SL2 alone reduced *sopB* expression in comparison to the wild type (Fig 5A). The presence of *csrAΔ*50 in this mutant of SL2, however, restored *sopB* expression to a level greater than that of the wild type. We next tested the functional interaction of CsrA with SL1. The effect of the *csrAΔ*50 mutant was, in fact, nearly identical to that of A25G mutant, with each increased more than 2-fold above the wild-type expression (Fig 5B). The combination of the two, however, was additive: the strain harboring both A25G and *csrAΔ*50 induced 4-fold more than the wild type. This result suggests both that, although CsrA binds to SL1 and requires the native sequence to do so efficiently, it continues to bind even to the weakened secondary structure, and that SL1 continues to repress invasion gene expression even in the absence of functional CsrA. We conversely tested the effects of increased CsrA activity using mutants of the BarA/SirA/Csr regulatory cascade. As expected, the loss of both *csrB* and *csrC* greatly reduced the expression of *sopB*, due to the likely abundance of free CsrA within the mutant strain (S5 Fig). The addition of the *hilD* A25G mutation to the *ΔcsrBC* mutant, however, greatly increased invasion gene expression, suggesting an inability of CsrA to efficiently interact with the altered *hilD* transcript. Similarly, the addition of the A25G mutation also enhanced *sopB* expression in a *sirA* mutant, but to a lesser degree, as would be expected as SirA is known to additionally induce invasion gene expression by means independent of CsrB and CsrC [25].

### Amplification of *hilD* overcomes the threshold for invasion gene induction

**Fig 6:**
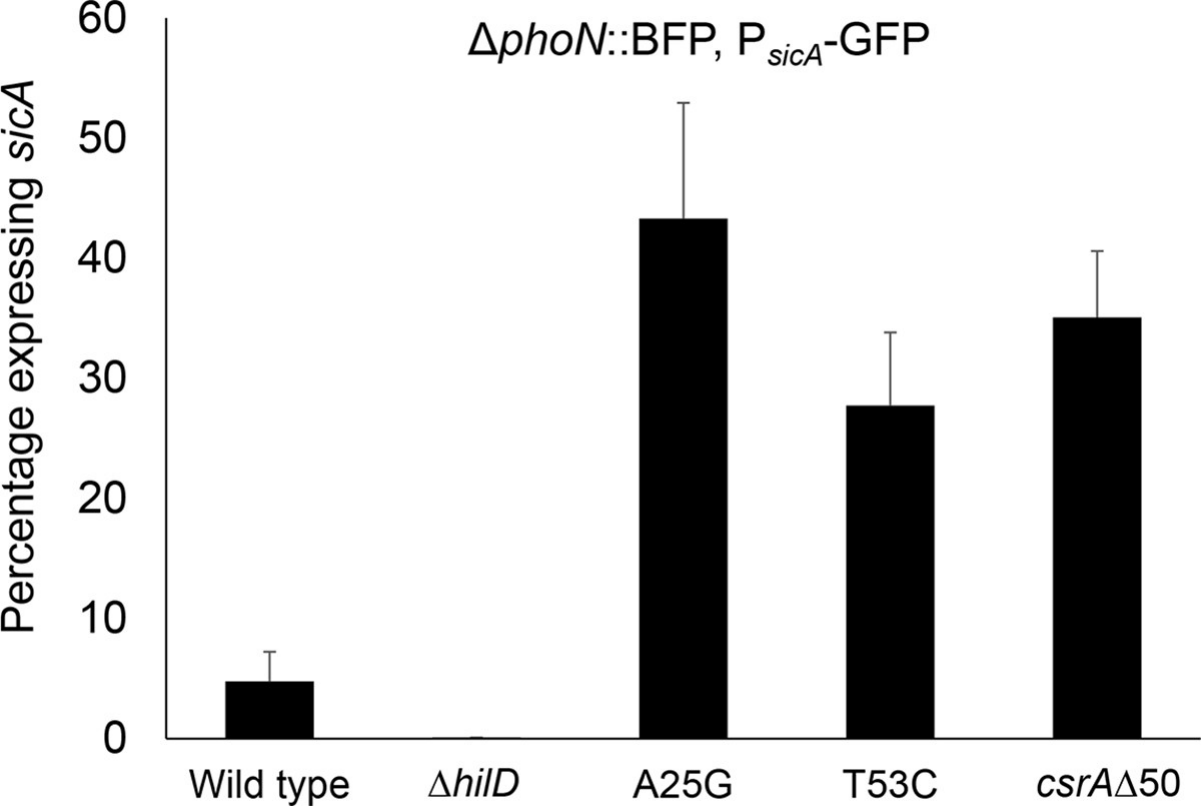
*hilD* message secondary structure and CsrA control the proportion of the invasion-competent *Salmonella* population. Strains constitutively expressing BFP were assessed for expression of the SPI-1 gene *sicA* using a *sicA*-GFP reporter fusion. The A25G and T53C mutants of *hilD* and the *csrAΔ*50 mutant demonstrated significantly increased *sicA* expression at P = 0.0002 or less. All strains were tested in triplicate; error bars show standard deviation.

Our results demonstrate that *hilD* message secondary together with CsrA elicit mean changes in invasion gene expression. Yet, *in vitro* and *in vivo*, *Salmonella* exists as two populations, one with invasion induced and the other not [3,26]. HilD acts to transcriptionally induce its own gene, allowing small changes in gene expression to produce disproportionately robust down-stream effects [27]. We thus tested whether the control of *hilD* by its RNA secondary structure creates a threshold effect, beyond which levels of HilD exert a self-perpetuating induction of invasion. The population dynamics of invasion were thus measured using GFP fused to the promoter of *sicA*, an invasion gene within SPI-1. We first ensured that HilD was required for this biphasic phenotype, as only 0.1% of the population produced measurable GFP in a *ΔhilD* null mutant, compared to 4.8% in the wild type strain (Fig 6 and S6 Fig). We further found that both SL1 and CsrA were required to regulate the biphasic expression of invasion genes, as mutations of either altered the ratio of the two populations. Indeed, both the A25G and T53C *hilD* mutants, with their reduced binding of CsrA to SL1, and the *csrAΔ*50 truncation mutant significantly increased the proportion of the invasion-competent population to 43%, 28% and 35%, respectively. These data thus demonstrate that CsrA and the stem-loop structure of *hilD*, while acting independently of the autoinduction that achieves biphasic expression, function to tune the proportion of invasion-competent bacteria.

## Discussion

**Fig 7:**
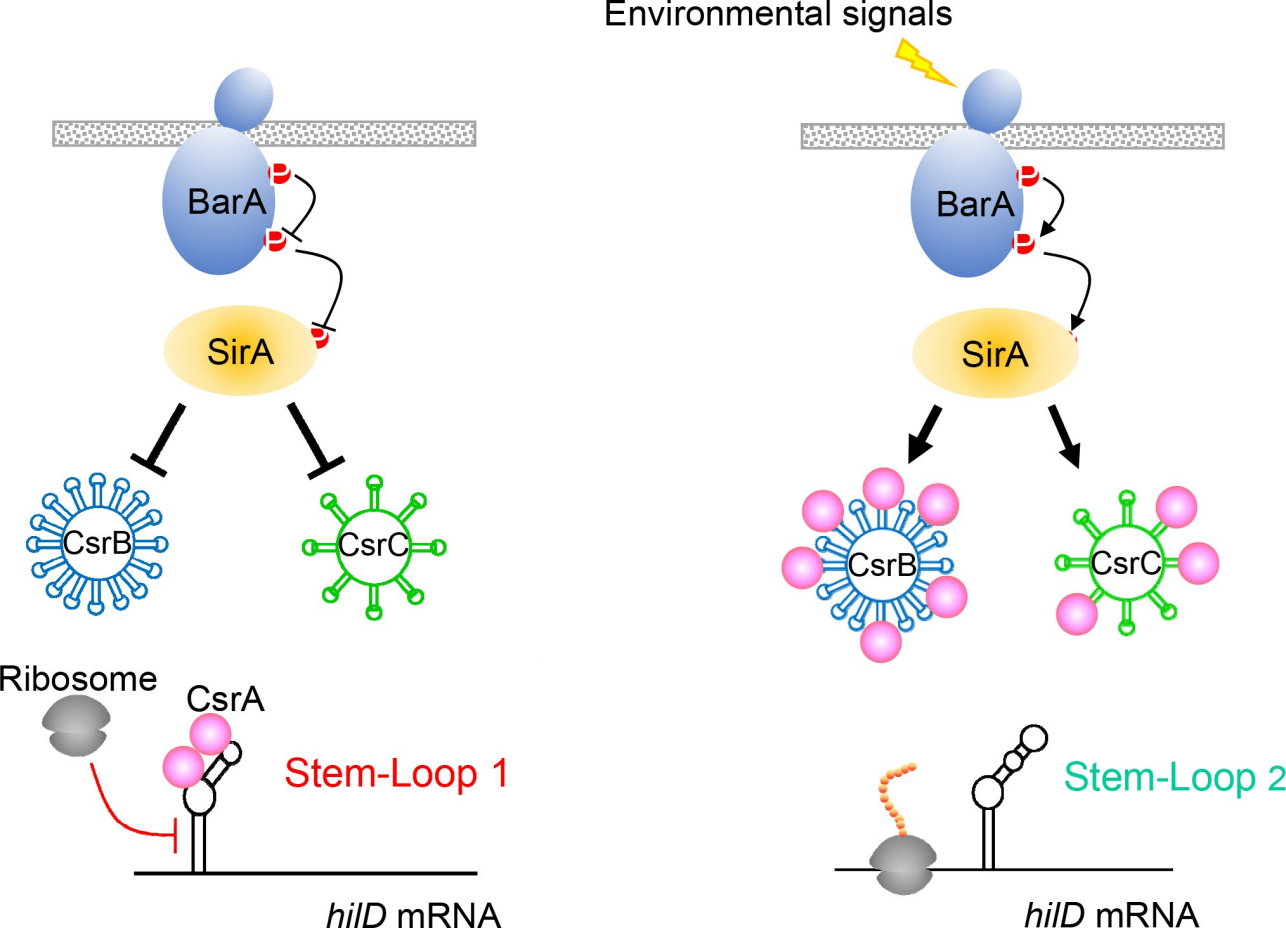
A model for the regulation of *Salmonella* invasion through the post-transcriptional control of *hilD*. Activation of the BarA/SirA two-component regulator induces transcription of two small RNAs, CsrB and CsrC. These bind multiple copies of CsrA, titrating it from its target, the *hilD* message. In the presence of free CsrA, the SL1 structure of the *hilD* message is stabilized by this regulatory protein, sequestering the ribosome-binding site and start codon, and thus hindering translation. When the concentration of free CsrA is reduced through its binding to CsrB and CsrC, the alternative *hilD* message secondary structure SL2 is formed, allowing translation of HilD and consequent expression of invasion genes.

Here we have described a sensitive mechanism for the control of *Salmonella* invasion, an essential virulence function. The data presented suggest a simple but elegant model (Fig 7): The message of the invasion activator HilD is capable of assuming two alternative and mutually exclusive secondary structures. Formation of the first, SL1, sequesters the ribosome-binding site and start codon, reducing message stability and presumably preventing translation. Formation of the second (SL2), however, liberates these sites from the secondary structure and instead promotes the expression of *hilD*. SL2 is energetically favored, and thus in the absence of additional regulatory components, it should predominate. SL1, however, binds to CsrA, further stabilizing it and shifting the balance of control towards the repression of *hilD* when in the presence of this global regulatory protein. This balance can thus be altered by the activity of the BarA/SirA two-component regulator, which induces the expression of the regulatory RNAs CsrB and CsrC. These RNAs bind CsrA, titrating it from its target within SL1, allowing SL2 to form and increasing invasion gene expression through enhanced translation of HilD.

The proposed system is thus comprised of elements familiar in the control of bacterial gene expression through mRNA regulation. Employing alternative secondary structures has long been recognized in bacteria as a means to alter translational efficiency [28]. In addition, CsrA is known to bind specific sequences within the context of message structure to reduce translation and repress gene expression [23]. The combination of these two therefore provides a sensitive means to switch between activation and repression. Of note is the fact that either a partial disruption of SL1 by mutation or the loss of functional CsrA increases invasion gene expression, but the effect of the two in combination remains additive (Fig 5B). Similarly, the combined mutation of CsrA and SL2 creates an intermediate phenotype (Fig 5A), as would be expected if the two were in competition. These findings thus demonstrate a fine balance in control, originating in the message but augmented by the regulatory protein.

Enteric pathogens such as *Salmonella* experience great environmental changes as they move into and through the intestinal tract of animals. The environmental cues that affect CsrA, and thus may tip the balance toward invasion, have been only partially elucidated. The activity of CsrA is reduced through its sequestration by two small RNAs, CsrB and CsrC, that are themselves induced by the BarA/SirA two-component regulator [15–17]. The signal for the BarA sensor-kinase has not been fully characterized, but CsrB and CsrC can be induced by acetate, independently of BarA, through the SirA response-regulator [29]. As acetate is a metabolic by-product of the intestinal microbiota and exists in millimolar concentrations in the gut, it provides at least one plausible signal for invasion induction through the system we describe. In addition to their sensitivity, changes in gene expression produced by mRNA switches are predicted to be rapid. The half-lives of bacterial messages are typically measured in minutes, such that changes in stability quickly alter the message pool available for translation. *Salmonella* controls invasion through multiple mechanisms, including transcription and protein activity. mRNA stability, however, is likely to provide a means to do so efficiently in response to the changing environmental conditions encountered by the pathogen.

As a pathogen, *Salmonella* must balance the costs of protein production with the expression of virulence factors needed to colonize an animal host. It has evolved to manage the costly invasion process by producing a biphasic population, with only a fraction burdened by the expression of invasion genes [3,26], but functioning to create an environment conducive to the proliferation of non-invasive siblings [19–22]. This implies the existence of an invasion switch with a specific set point, and one that can be altered by genetic and environmental cues. The system described here, in conjunction with the auto-induction of HilD, constitutes an important component of such a switch. Invasion requires a complex interplay of transcriptional regulators, with HilD and others comprising a feed-forward induction loop. The fine control of the *hilD* message level may thus provide a threshold for induction: The small proportion of the population with sufficient *hilD* message to produce HilD and induce subsequent autoinduction are invasive, while those that do not reach that threshold are not. In this way, the switch described here may play an important role to produce the two distinct populations needed for virulence.

## Materials and methods

### Ethics statement

Studies involving vertebrate animals were approved by the Cornell University Institutional Animal Care and Use Committee (Protocol 2012–0074). Euthanasia was conducted using carbon dioxide inhalation in accordance with the American Veterinary Medical Association Guidelines for Euthanasia of Animals. The Cornell University Animal Care and Use program and associated animal facilities are operated in accordance with the U.S. Department of Agriculture Animal Welfare Act (1966), Regulation (C.F.R., 2009) and policies, the Health Research Extension Act (1985), the Public Health Service Policy on Humane Care and Use of Laboratory Animals (PHS, 2002), the Guide for the Care and Use of Laboratory Animals (NRC, 2011), the Guide for the Care and Use of Agricultural Animals in Research and Teaching (2010), the New York State Health Law (Article 5, Section 504), and other applicable federal, state, and local laws, regulations, policies, and guidelines.

### Bacterial strains, plasmids and growth

Bacterial strains and plasmids are listed in S1 Table. Chromosomal point mutants were made using CRISPR/Cas9-directed mutagenesis as described below. Strains were grown in LB broth (10 g tryptone, 5 g yeast extract, 5 g NaCl/liter) with 100 mM MOPS pH 6.7 at 37°C unless otherwise stated.

### Screen for *hilD* mutants

The *hilD* ORF and a portion of its 5’UTR extending to the transcriptional start site were amplified using error-prone PCR to create point mutations, and PCR products were cloned into a derivative of pWSK29 to create pWSK29-*tetRA*-*hilD*-3XFLAG, placing *hilD* under the control of the inducible *tetA* promoter (S1 Fig). The plasmid library was transformed into a *ΔhilD*, *sipC*::*gfp* strain, selected on LB agar with 100 mM MOPS pH 6.7, 100 μg/ml ampicillin, and 1 mM sodium nonanoate. Tetracycline was not included in the medium as basal expression of the *tetA* promoter was adequate to express *hilD* from the multi-copy plasmid in its absence. Bacteria expressing GFP under these repressive conditions were identified by green fluorescence using an Olympus OV-100 imaging system. Resulting plasmids were sequenced for mutations in *hilD* and the adjacent untranslated region.

### Reporter fusion assays

For β-galactosidase assays, strains carrying *lacZY* fusions were grown as 5 ml cultures in 18 mm glass tubes without aeration for 16–18 hours. Assays were performed as previously described [30]. Luminescence assays using *luxCDABE* fusions were conducted as described [31]. Briefly, strains were grown overnight in the presence of tetracycline (25 μg/ml) for plasmid maintenance and then diluted 100-fold in the same medium. Samples of 150 μl were inoculated into 96-well plates, and luminescence and OD_600_ were read every 20 minutes for 24 hours using a Synergy 2 luminescence microplate reader (BioTek). Samples were tested in replicates of four or more.

### RT-PCR

Strains containing a plasmid-borne *hilD-*3xFLAG under *tetA* promoter control, with additional point mutations as indicated and chromosomal *hilD* deleted, were grown overnight standing in LB with 100 mM MOPS pH 6.7. After 16.5 hr, 1 ml of each culture was collected and washed once in PBS. RNA was extracted with phenol:chloroform, and Turbo DNase (Ambion) was used to reduce contaminating DNA. SuperScript II RTase (Invitrogen) was used to synthesize cDNA, which served as template for RT-qPCR reactions using iQ Sybr Green Reagent. The *ΔΔ*Ct method was used to calculate the amount of *hilD* transcript relative to the housekeeping gene *dnaN*.

### Construction of *hilD* chromosomal mutants

Mutants were constructed using the previously reported plasmid-based CRISPR/Cas system [32]. Plasmids required for mutant construction are listed in S1 Table and oligonucleotide sequences in S2 Table. In brief, the pCRISPR::*hilD* plasmids carrying the sequence targeting a specific PAM site within *hilD* were created by phosphorylating a pair of synthesized oligonucleotides, annealing, and cloning into the *Bsa*I sites of pCRISPR (Addgene). The resulting plasmid was co-transformed along with a synthesized single-stranded oligonucleotide carrying the desired point mutation of *hilD* into a *Salmonella* strain carrying the plasmid pKD46 [33], producing the Red λ recombinase, and pCas9 (Addgene) expressing tracrRNA and Cas9. The transformants were initially selected on LB agar with chloramphenicol (25 μg/ml) and kanamycin (50 μg/ml) at 37°C. Colonies were purified once onto the LB agar, and further screened for loss of pCRISPR::*hilD* and pCas9 plasmids by susceptibility to chloramphenicol and kanamycin.

### Cell invasion assays

HEp-2 cells were cultured in DMEM containing 10% fetal bovine serum. For invasion assays, 2 x 10^5^ cells were seeded in 24-well plates. Bacteria were grown overnight as static cultures in LB with 100 mM HEPES, pH 8.0, at 37°C. To maintain an MOI of 10, ~2 x 10^6^ bacteria were added to cells. Plates were then centrifuged for 10 min at 100 x *g* and incubated for 1 h at 37°C in 95% air/5% CO_2_. Media was removed, the cells were washed four times with HBSS, followed by incubation with media supplemented with 20 μg/ml gentamicin for 1 h. Post incubation, cells were washed three times with sterile PBS and lysed with 1% Triton X-100 for 5 min. Intracellular bacteria were quantified by dilution of lysates onto LB agar. Invasion was determined by dividing recovered bacteria by its inoculum. Each strain was tested in quadruplicate wells in each of at least two independent experiments.

### Animal experiments

Strains were grown overnight, washed twice and resuspended in PBS. Differently marked strain pairs were mixed in approximately equal proportions: a *malXY*::kan strain (kanamycin-resistant) with an A25G, T53C, *malXY*::cam strain (chloramphenicol-resistant), or a *malXY*::cam strain with an A25G, T53C *malXY*::kan strain, to compensate for any effects of the extraneous resistance marker. Female BALB/c mice, 6- to 7-weeks of age, were given ~1x10^7^ total bacteria (5x10^6^ of each strain) by mouth. After five days, mice were euthanized and the spleens and livers were homogenized in PBS, with dilutions plated onto LB agar with 25 μg/ml chloramphenicol or 100 μg/ml kanamycin. Data were pooled to determine a competitive index, the ratio of the wild type to the A25G, T53C mutant.

### CsrA binding assays

CsrA protein was expressed as a carboxyl-terminal his-tagged construct using the pQE70 expression vector (Qiagen) and purified using affinity chromatography. Protein was mixed at a concentration of 100 nM with 50 nM of 5’-biotinylated RNA probes (Sigma) in binding buffer (10 mM Tris pH 7.5, 10mM MgCl_2_, 100 mM KCl, 7.5% glycerol, 20 mM DTT and 0.01% sodium deoxycholate) for 35 minutes at 37°C. Reactions were spotted in replicates of five onto 0.2 μm nitrocellulose membrane, air dried and UV crosslinked at 120 μJ/cm^2^. Membranes were blocked for one hour in TBS SuperBlock (Thermo Scientific), incubated with 1:50,000 diluted streptavidin-conjugated horseradish peroxidase and developed with Western Lightning ECL Pro chemiluminescence substrate (Perkins Elmer). ImageJ was used to quantify intensity.

### Flow cytometry

Strains expressing a constitutive BFP (for gating on the *Salmonella* population), a GFP reporter downstream of the *sicA* promoter (for assessing invasion gene expression) [34], and additional chromosomal mutations as indicated, were grown overnight. After 16.5 hr, 100 μl to 1 ml of each culture was fixed in 4% paraformaldehyde in PBS, turning at 4°C for 30 min. Samples were centrifuged, paraformaldehyde was aspirated, and fixed bacteria were resuspended in PBS. Samples were analyzed on a FACS Aria III Custom, gating on the blue population and interrogating for GFP-expressing bacteria.

### Statistics

Comparisons of means were performed with Student’s *t*-test using JMP 11 Pro.

## Supporting information

S1 Table Strains and plasmids. (DOCX)

S2 Table Oligonucleotides used in mutant construction. (DOCX)

S1 Fig Screen for gain-of-function mutants of hilD. (*A*) *hilD* was expressed under the control of a tetracycline-inducible *tetA* promoter and carried a 3’ 3x-FLAG tag for immunoblotting. (*B*) Nonanoate represses invasion gene expression. Strains were grown with 1 mM sodium nonanoate or no additive and assessed for invasion gene expression using a single-copy chromosomal *hilA*::*lacZ* fusion by β-galactosidase assays. (*C*) Nonanoate reduces the stability of the HilD protein. Protein production in cultures of bacteria grown with or without nonanoic acid was halted using an antibiotic mixture, and HilD was measured at subsequent time points by blotting with an anti-3x-FLAG tag antibody. (*D*) Screening method for *hilD* mutants. Error-prone PCR was used to generate mutations within the *hilD* ORF and upstream region extending to the transcriptional start site. PCR products were then cloned into a plasmid vector, placing *hilD* under the control of a tetracycline-inducible promoter. The plasmid library so generated was transformed into a Δ*hilD*, *sipC*::GFP strain, and selected on LB agar buffered to pH 6.7 with 100 mM MOPS, 1 mM sodium nonanoate, and 100 μg/ml ampicillin (for plasmid selection), and colonies were screened for green fluorescence.
(DOCX)

S2 Fig Mutations identified within the 5’ untranslated region and extending into the 5’ end of the hilD ORF. Sequence of the region is shown at the top, with mutated nucleotides identified by the screen shown in red. Rows of sequence indicate specific clones identified, with changes shown in red. The locations of the transcriptional start site (+1), the *hilD* ORF, and the inverted repeats that form the stem portion of SL1 (stem-loop) are shown.
(DOCX)

S3 Fig Mutations affecting the hilD message secondary structure alter hilD expression and bacterial growth. (*A*) Expression of *hilD* was measured over time using a *luxCDABE* transcriptional reporter fusion (n = 5), assessing luminescence normalized to bacterial numbers (luminescence/OD_600_). All strains differed from the wild type for mean peak expression at P < 0.0001. (*B*) Mutations of the *hilD* message that disrupt SL1 and induce invasion gene expression reduce bacterial growth rate. Bacteria were grown for 24 hours and growth was measured using OD_600_.
(DOCX)

S4 Fig Mutations of the hilD transcript predicted to reduce SL1 stability do not reduce Salmonella colonization of liver or spleen. BALB/c mice (*Slc11a1*^-/-^) were inoculated orally with wild type, *hilD* A25G or T53C mutant strains, and colony-forming units (cfu) cultured from spleens and livers four days after infection were counted (n = 5 for each strain). Box plots are defined by the upper and lower quartile, with median shown by the horizontal line. Whiskers show maximum and minimum values. All strains carried a *phoN*::*kan* insertion for selection on kanamycin. Neither mutant demonstrated organ infection significantly different from that of the wild type.
(DOCX)

S5 Fig *hilD* message secondary structure affects the control of invasion gene expression in mutants of the BarA/SirA/Csr regulatory cascade. Expression of the invasion gene *sopB* was determined in the strains shown using a *luxCDABE* transcriptional reporter fusion and measuring luminescence normalized to bacterial numbers (luminescence/OD_600_). Repression of *sopB* expression due to the loss of the small RNAs CsrB and CsrC or the response-regulator SirA was abrogated by the *hilD* A25G mutation that disrupts SL1. Data show mean ±SD (n = 5 for each strain).
(DOCX)

S6 Fig Representative flow cytometry data. Strains constitutively expressing BFP (Δ*phoN*::BFP) were assessed for expression of the SPI-1 gene *sicA* using a *sicA*-GFP reporter fusion (P_*sicA*_-GFP). The wild type strain, without fluorescent protein genes, was used to establish the gating threshold for BFP, and the Δ*phoN*::BFP, P_*sicA*_-GFP strain was used to determine the gating threshold for the biphasic GFP signal. Peaks on the right show the portion of the population expressing GFP; those on the left show that without detectable GFP expression.
(DOCX)
