# Supplementary material for: Salmonella invasion is controlled through the secondary structure of the hilD transcript
Source: PLoS Pathog. 2019 Apr 24;15(4):e1007700. doi: 10.1371/journal.ppat.1007700 (PMC6502421; doi:10.1371/journal.ppat.1007700)
Supplement: S1 Table — (DOCX) [file ppat.1007700.s001.docx]

| **Strain** | **Relevant Genotype** | **Source/Reference** |
| --- | --- | --- |
| *Salmonella* Typhimurium 14028s | Wild type | ATCC |
| CA3717 | Δ*hilD*, *sipC*::GFP | This study |
| CA4264 | *hilD* A25G | This study |
| CA4273 | *hilD* C50T | This study |
| CA4271 | *hilD* T53C | This study |
| CA4277 | *hilD* A25G, T53C | This study |
| CA4296 | *hilD* A57T, G58C, T59C | This study |
| CA412 | *sipC::lacZY* | Bajaj, 1996 |
| CA4278 | *hilD* A25G, *sipC::lacZY* | This study |
| CA4280 | *hilD* C50T, *sipC::lacZY* | This study |
| CA4279 | *hilD* T53C, *sipC::lacZY* | This study |
| CA4282 | *hilD* A25G, T53C, *sipC::lacZY* | This study |
| CA2285 | Wild type, pBA409 | Hung, 2013 |
| CA4283 | *hilD* A25G, pBA409 | This study |
| CA4286 | *hilD* C50T, pBA409 | This study |
| CA4285 | *hilD* T53C, pBA409 | This study |
| CA4288 | *hilD* A25G, T53C, pBA409 | This study |
| CA4331 | *hilD* A57T, G58C, T59C, pBA409 | This study |
| CA4306 | *csrA*Δ50, pBA409 | This study |
| CA4389 | *hilD* A25G, *csrA*Δ50, pBA409 | This study |
| CA4380 | *hilD* A57T, G58C, T59C, *csrA*Δ50, pBA409 | This study |
| CA4772 | Δ*csrBC*, pBA409 | This study |
| CA4771 | *hilD* A25G, Δ*csrBC*, pBA409 | This study |
| CA4769 | Δ*sirA*, pBA409 | This study |
| CA4767 | *hilD* A25G, Δ*sirA*, pBA409 | This study |
| CA2285 | Wild type, pBA427 | Hung, 2013 |
| CA4339 | *hilD* A25G, pBA427 | This study |
| CA4347 | *hilD* C50T, pBA427 | This study |
| CA4343 | *hilD* T53C, pBA427 | This study |
| CA4340 | *hilD* A25G, T53C, pBA427 | This study |
| CA4314 | *hilD* A57T, G58C, T59C, pBA427 | This study |
| CA4436 | *malXY*::kan | This study |
| CA4434 | *malXY*::cam | This study |
| CA4440 | *hilD* A25G, T53C, *malXY*::kan | This study |
| CA4438 | *hilD* A25G, T53C, *malXY*::cam | This study |
| CA4129 | Δ*hilD*, *sipC::lacZY*, pCA211 | This study |
| CA4130 | Δ*hilD*, *sipC::lacZY*, pCA217 | This study |
| CA4131 | Δ*hilD*, *sipC::lacZY*, pCA218 | This study |
| CA4132 | Δ*hilD*, *sipC::lacZY*, pCA219 | This study |
| CA4217 | Δ*hilD*, *sipC::lacZY*, pCA225 | This study |
| CA3887 | Δ*phoN*::BFP, P*_sicA_*-GFP | Eade, 2018 |
| CRE208 | Δ*phoN*::BFP, P*_sicA_*-GFP, *csrA*Δ50 | This study |
| CRE186 | Δ*phoN*::BFP, P*_sicA_*-GFP, *hilD* A25G | This study |
| CA4745 | Δ*phoN*::BFP, P*_sicA_*-GFP, *hilD* T53C | This study |
| CA4178 | Δ*phoN*::BFP, P*_sicA_*-GFP, Δ*hilD* | This study |
| CA4294 | *E. coli* SG10039, pCA144 | This study |
| **Plasmid** |  |  |
| pWSK29-*tetRA*-*hilD*-3XFLAG | P*_tetA_*-*hilD*-3XFLAG | This study |
| pBA409 | *sopB::luxCDABE* | Hung, 2013 |
| pBA427 | *hilD::luxCDABE* | Hung, 2013 |
| pCA211 | P*_tetA_*-*hilD* | This study |
| pCA217 | P*_tetA_*-*hilD* A25G | This study |
| pCA218 | P*_tetA_*-*hilD* T53C | This study |
| pCA219 | P*_tetA_*-*hilD* A25G, T53C | This study |
| pCA225 | P*_tetA_*-*hilD* C50T | This study |
| pCA144 | pQE70-*csrA* | This study |
| pCRISPR | Cloning of pCRISPR::*hilD* constructs | Addgene |
| pCas9 | tracrRNA and Cas9 | Addgene |
