# Supplementary material for: Salmonella invasion is controlled through the secondary structure of the hilD transcript
Source: PLoS Pathog. 2019 Apr 24;15(4):e1007700. doi: 10.1371/journal.ppat.1007700 (PMC6502421; doi:10.1371/journal.ppat.1007700)
Supplement: S2 Table — (DOCX) [file ppat.1007700.s002.docx]

| **PAM site-containing oligonucleotide pairs** | **Targeted mutation** |
| --- | --- |
| AAACCAGTAAGGAACATTAAAATAACATCAACAAG  AAAACTTGTTGATGTTATTTTAATGTTCCTTACTG | A25G |
| AAACCGGCAGGACGCTGATGACTATTACTTACAAG  AAAACTTGTAAGTAATAGTCATCAGCGTCCTGCCG | C50T  T53C  A57T,G58C,T59C |
| **Mutagenic oligonucleotides** | **Mutant created** |
| CAGTAAGGAACATTAAAATAACATCAACGAAGGGATAATATGGAAAATGTAACCTTTGTA | A25G |
| AACAAAGGGATAATATGGAAAATGTAACTTTTGTAAGTAATAGTCATCAGCGTCCTGCCG | C50T |
| AACAAAGGGATAATATGGAAAATGTAACCTTCGTAAGTAATAGTCATCAGCGTCCTGCCG | T53C |
| CATCAACAAAGGGATAATATGGAAAATGTAACCTTTGTATCCAATAGTCATCAGCGTCCTGCCGCAGATAACTTACAGAA | A57T,G58C,T59C |
