# Supplementary material for: Salmonella invasion is controlled through the secondary structure of the hilD transcript
Source: PLoS Pathog. 2019 Apr 24;15(4):e1007700. doi: 10.1371/journal.ppat.1007700 (PMC6502421; doi:10.1371/journal.ppat.1007700)
Supplement: S1 Fig — (A) hilD was expressed under the control of a tetracycline-inducible tetA promoter and carried a 3’ 3x-FLAG tag for immunoblotting. (B) Nonanoate represses invasion gene expression. Strains were grown with 1 mM sodium nonanoate or no additive and assessed for invasion gene expression using a single-copy chromosomal hilA::lacZ fusion by β-galactosidase assays. (C) Nonanoate reduces the stability of the HilD protein. Protein production in cultures of bacteria grown with or without nonanoic acid was halted using an antibiotic mixture, and HilD was measured at subsequent time points by blotting with an anti-3x-FLAG tag antibody. (D) Screening method for hilD mutants. Error-prone PCR was used to generate mutations within the hilD ORF and upstream region extending to the transcriptional start site. PCR products were then cloned into a plasmid vector, placing hilD under the control of a tetracycline-inducible promoter. The plasmid library so generated was transformed into a ΔhilD, sipC::GFP strain, and selected on LB agar buffered to pH 6.7 with 100 mM MOPS, 1 mM sodium nonanoate, and 100 μg/ml ampicillin (for plasmid selection), and colonies were screened for green fluorescence. (DOCX) [file ppat.1007700.s003.docx]

**S1 Fig. Screen for gain-of-function mutants of *hilD*.** (*A*) *hilD* was expressed under the control of a tetracycline-inducible *tetA* promoter and carried a 3’ 3x-FLAG tag for immunoblotting. (*B*) Nonanoate represses invasion gene expression. Strains were grown with 1 mM sodium nonanoate or no additive and assessed for invasion gene expression using a single-copy chromosomal *hilA::lacZ* fusion by β-galactosidase assays. (*C*) Nonanoate reduces the stability of the HilD protein. Protein production in cultures of bacteria grown with or without nonanoic acid was halted using an antibiotic mixture, and HilD was measured at subsequent time points by blotting with an anti-3x-FLAG tag antibody. (*D*) Screening method for *hilD* mutants. Error-prone PCR was used to generate mutations within the *hilD* ORF and upstream region extending to the transcriptional start site. PCR products were then cloned into a plasmid vector, placing *hilD* under the control of a tetracycline-inducible promoter. The plasmid library so generated was transformed into a Δ*hilD*, *sipC*::GFP strain, and selected on LB agar buffered to pH 6.7 with 100 mM MOPS, 1 mM sodium nonanoate, and 100 μg/ml ampicillin (for plasmid selection), and colonies were screened for green fluorescence.
