# Supplementary material for: Salmonella invasion is controlled through the secondary structure of the hilD transcript
Source: PLoS Pathog. 2019 Apr 24;15(4):e1007700. doi: 10.1371/journal.ppat.1007700 (PMC6502421; doi:10.1371/journal.ppat.1007700)
Supplement: S2 Fig — Sequence of the region is shown at the top, with mutated nucleotides identified by the screen shown in red. Rows of sequence indicate specific clones identified, with changes shown in red. The locations of the transcriptional start site (+1), the hilD ORF, and the inverted repeats that form the stem portion of SL1 (stem-loop) are shown. (DOCX) [file ppat.1007700.s004.docx]

**S2 Fig. Mutations identified within the 5’ untranslated region and extending into the 5’ end of the *hilD* ORF.** Sequence of the region is shown at the top, with mutated nucleotides identified by the screen shown in red. Rows of sequence indicate specific clones identified, with changes shown in red. The locations of the transcriptional start site (+1), the *hilD* ORF, and the inverted repeats that form the stem portion of SL1 (stem-loop) are shown.
