# Supplementary material for: Salmonella invasion is controlled through the secondary structure of the hilD transcript
Source: PLoS Pathog. 2019 Apr 24;15(4):e1007700. doi: 10.1371/journal.ppat.1007700 (PMC6502421; doi:10.1371/journal.ppat.1007700)
Supplement: S3 Fig — (A) Expression of hilD was measured over time using a luxCDABE transcriptional reporter fusion (n = 5), assessing luminescence normalized to bacterial numbers (luminescence/OD600). All strains differed from the wild type for mean peak expression at P < 0.0001. (B) Mutations of the hilD message that disrupt SL1 and induce invasion gene expression reduce bacterial growth rate. Bacteria were grown for 24 hours and growth was measured using OD600. (DOCX) [file ppat.1007700.s005.docx]

**S3 Fig. Mutations affecting the *hilD* message secondary structure alter *hilD* expression and bacterial growth.** (*A*) Expression of hilD was measured over time using a *luxCDABE* transcriptional reporter fusion (n=5), assessing luminescence normalized to bacterial numbers (luminescence/OD_600_). All strains differed from the wild type for mean peak expression at P < 0.0001. (*B*) Mutations of the *hilD* message that disrupt SL1 and induce invasion gene expression reduce bacterial growth rate. Bacteria were grown for 24 hours and growth was measured using OD_600_.
