# Supplementary material for: Salmonella invasion is controlled through the secondary structure of the hilD transcript
Source: PLoS Pathog. 2019 Apr 24;15(4):e1007700. doi: 10.1371/journal.ppat.1007700 (PMC6502421; doi:10.1371/journal.ppat.1007700)
Supplement: S4 Fig — BALB/c mice (Slc11a1-/-) were inoculated orally with wild type, hilD A25G or T53C mutant strains, and colony-forming units (cfu) cultured from spleens and livers four days after infection were counted (n = 5 for each strain). Box plots are defined by the upper and lower quartile, with median shown by the horizontal line. Whiskers show maximum and minimum values. All strains carried a phoN::kan insertion for selection on kanamycin. Neither mutant demonstrated organ infection significantly different from that of the wild type. (DOCX) [file ppat.1007700.s006.docx]

**S4 Fig. Mutations of the *hilD* transcript predicted to reduce SL1 stability do not reduce *Salmonella* colonization of liver or spleen.** BALB/c mice (*Slc11a1*^-/-^) were inoculated orally with wild type, *hilD* A25G or T53C mutant strains, and colony-forming units (cfu) cultured from spleens and livers four days after infection were counted (n=5 for each strain). Box plots are defined by the upper and lower quartile, with median shown by the horizontal line. Whiskers show maximum and minimum values. All strains carried a *phoN::kan* insertion for selection on kanamycin. Neither mutant demonstrated organ infection significantly different from that of the wild type.
