# Supplementary material for: Salmonella invasion is controlled through the secondary structure of the hilD transcript
Source: PLoS Pathog. 2019 Apr 24;15(4):e1007700. doi: 10.1371/journal.ppat.1007700 (PMC6502421; doi:10.1371/journal.ppat.1007700)
Supplement: S5 Fig — Expression of the invasion gene sopB was determined in the strains shown using a luxCDABE transcriptional reporter fusion and measuring luminescence normalized to bacterial numbers (luminescence/OD600). Repression of sopB expression due to the loss of the small RNAs CsrB and CsrC or the response-regulator SirA was abrogated by the hilD A25G mutation that disrupts SL1. Data show mean ±SD (n = 5 for each strain). (DOCX) [file ppat.1007700.s007.docx]

**S5 Fig. *hilD* message secondary structure affects the control of invasion gene expression in mutants of the BarA/SirA/Csr regulatory cascade.** Expression of the invasion gene *sopB* was determined in the strains shown using a *luxCDABE* transcriptional reporter fusion and measuring luminescence normalized to bacterial numbers (luminescence/OD_600_). Repression of *sopB* expression due to the loss of the small RNAs CsrB and CsrC or the response-regulator SirA was abrogated by the *hilD* A25G mutation that disrupts SL1. Data show mean ±SD (n=5 for each strain).
