# Supplementary material for: Salmonella invasion is controlled through the secondary structure of the hilD transcript
Source: PLoS Pathog. 2019 Apr 24;15(4):e1007700. doi: 10.1371/journal.ppat.1007700 (PMC6502421; doi:10.1371/journal.ppat.1007700)
Supplement: S6 Fig — Strains constitutively expressing BFP (ΔphoN::BFP) were assessed for expression of the SPI-1 gene sicA using a sicA-GFP reporter fusion (PsicA-GFP). The wild type strain, without fluorescent protein genes, was used to establish the gating threshold for BFP, and the ΔphoN::BFP, PsicA-GFP strain was used to determine the gating threshold for the biphasic GFP signal. Peaks on the right show the portion of the population expressing GFP; those on the left show that without detectable GFP expression. (DOCX) [file ppat.1007700.s008.docx]

**S6 Fig. Representative flow cytometry data.** Strains constitutively expressing BFP (Δ*phoN*::BFP) were assessed for expression of the SPI-1 gene *sicA* using a *sicA*-GFP reporter fusion (P*_sicA_*-GFP). The wild type strain, without fluorescent protein genes, was used to establish the gating threshold for BFP, and the Δ*phoN*::BFP, P*_sicA_*-GFP strain was used to determine the gating threshold for the biphasic GFP signal. Peaks on the right show the portion of the population expressing GFP; those on the left show that without detectable GFP expression.
